# Basal and Spasmolytic Effects of a Hydroethanolic Leaf Extract of *Melissa officinalis* L. on Intestinal Motility: An *Ex Vivo* Study

**DOI:** 10.1089/jmf.2018.0154

**Published:** 2019-07-10

**Authors:** Philippe Aubert, Isabelle Guinobert, Claude Blondeau, Valérie Bardot, Isabelle Ripoche, Pierre Chalard, Michel Neunlist

**Affiliations:** ^1^ Bretagne Loire University, Nantes University, INSERM 1235, IMAD, The Enteric Nervous System in Gut and Brain Disorders, Nantes, France.; ^2^ PiLeJe Group, Paris, France.; ^3^ Naturopôle, Saint-Bonnet de Rochefort, France.; ^4^ Clermont Auvergne University, CNRS, SIGMA Clermont, Clermont-Ferrand Chemistry Institute, Clermont Ferrand, France.

**Keywords:** hydroethanolic extract, intestine, lemon balm, rosmarinic acid

## Abstract

*Melissa officinalis* L. (lemon balm) has been used for decades with symptomatic benefits in patients with digestive disorders. However, very little is known on the effects of *M. officinalis* on the gastrointestinal (GI) tract. In this study, the basal and spasmolytic properties of a hydroethanolic leaf extract (HLE) of *M. officinalis* were assessed *ex vivo* on different segments of the GI tract of mice after phytochemical characterization of the extract. *M. officinalis* HLE had site- and dose-dependent effects on the contractile activity of the GI tract, the motility response being impacted in the jejunum and ileum but not in the antrum and colon. The observed effects could be caused by the phenolic compounds (mainly rosmarinic acid) detected in the extract.

## Introduction

*Melissa officinalis* L., also known as lemon balm, is a medicinal plant that has long been used in traditional medicine for digestive comfort and its impact upon mood and cognitive performance.^1–3^ As for gastrointestinal (GI) function, the European Medicines Agency acknowledged *M. officinalis* folium as a traditional herbal medicinal product for the symptomatic treatment of mild GI complaints including bloating and flatulence,^4^ and the European Scientific Cooperative On Phytotherapy, for the symptomatic treatment of digestive disorders such as minor spasms.^5^

Although *M. officinalis* has been used for decades with symptomatic benefits in patients with digestive disorders, to date, little is known on the effects of *M. officinalis* on the GI tract. The pathophysiological concepts concerning functional disorders of the GI tract include disturbances in motility and acid production as underlying mechanisms. Results of the few studies that have been performed to characterize the effects of *M. officinalis*, mainly on motility, are scarce and often contradictory. Most of the studies available were performed with *M. officinalis* folium extract that is a constituent of a marketed fixed combination of nine herbal extracts.^6–10^ The observations made in these studies were dependent on the gut part tested and were in part species dependent. For instance, Forster *et al.* did not show spasmolytic activity of *M. officinalis* in the guinea pig ileum while Heinle *et al.* observed a reduction in histamine-induced contractile response.^6,7^ Furthermore, under basal conditions, Schemann *et al.* reported increased motility in proximal and distal stomach while Sibaev *et al.* observed lemon balm leaf had no effects in basic electrophysiological properties of smooth muscle cells in the ileum.^8,9^ Altogether, no comprehensive studies on the effects of *M. officinalis* hydroethanolic leaf extracts (HLEs) upon basal contractile activity and spasmolytic activity in various gut segments have been performed. Therefore, the basal and spasmolytic properties of HLE of *M. officinalis* were assessed *ex vivo* on different segments of the GI tract of mice after phytochemical characterization of the extract.

## Materials and Methods

*M. officinalis* leaves were collected in Aubiat (France) in June 2015 and identified by Gilles Thébault from the herbarium of Museum d'Histoire Naturelle Henri-Lecoq (Clermont-Ferrand, France) where a voucher specimen was deposited (CLF106452). This herbarium is referenced with the international association for plant taxonomy whose head office is in the New York botanical garden.

### Preparation of *M. officinalis* HLE

*M. officinalis* HLE was produced by PiLeJe Industrie (France) according to the patented process WO2001056584A1. The procedure was standardized and the extract quality was controlled by thin layer chromatography, and by high-performance liquid chromatography (HPLC) for determination of rosmarinic acid content as described in the European Pharmacopeia monograph (ref 01/2011:1447). *M. officinalis* fresh frozen leaves were extracted with 20% to 70% (v/v) ethanolic leaching. The extract was concentrated under reduced pressure to evaporate ethanol and then ethanol concentration was adjusted to 30%. *M. officinalis* HLE in ethanol was used in *ex vivo* experiments. The specific batch used in the study (no. C-14J297) contained 8.6% of dry material that held 5.3% of rosmarinic acid. The yield of extraction as a percentage weight of the dry matter of starting fresh plant material was 24%. After addition of glycerol, the *M. officinalis* HLE is a rosmarinic acid standardized extract of *M. officinalis* (EPS Mélisse; PiLeJe Laboratoire, France).

### Phytochemical analysis of *M. officinalis* HLE

Chemical composition of *M. officinalis* HLE was further analyzed using ultra-HPLC (UHPLC). Chromatographic analyses by UHPLC were performed on an Ultimate 3000 RSLC UHPLC system (Thermo Fisher Scientific, Inc., MA, USA) coupled to a quaternary rapid separation pump (Ultimate autosampler) and a rapid separation diode array detector. Compounds were separated on a Uptisphere Strategy C18 column (250 × 4.6 mm, 5 *μ*m; Interchim), which was controlled at 30°C. The mobile phase was a mixture of 0.1% (v/v) formic acid in water (phase A) and 0.1% (v/v) formic acid in acetonitrile (phase B). The gradient of phase A was 100% (0–39 min), 0% (40–49 min), 100% (50–60 min), and then was held at 100%. The flow rate was 0.8 mL/min, and the injection volume was 5 *μ*L. The UHPLC system was connected to an Orbitrap (Thermo Fisher Scientific, Inc.) mass spectrometer, operated in negative electrospray ionization mode. Source operating conditions were as follows: 3 kV spray voltage; 320°C heated capillary temperature; 400°C auxiliary gas temperature; sheath, sweep, and auxiliary gas (nitrogen) flow rate of 50, 10 and 2 arbitrary units, respectively; and collision cell voltage between 10 and 50 eV. Full scan data were obtained at a resolution of 70,000, whereas tandem mass spectrometry (MS^2^) data were obtained at a resolution of 17,500. Data were processed using Xcalibur software (Thermo Fisher Scientific, Inc.).

Compounds detected were characterized according to their retention times, mass spectral data, and comparison with authentic standards when available or with data found in the literature.^3^

### Effects of *M. officinalis* HLE on GI motility

Male C57BL/6J Rj mice aged 8 weeks (*n* = 40; Janvier Laboratory, Le Genest-Saint-Isle, France) were maintained on a 12 h light–12 h dark cycle (22°C) and had free access to food and water. After 1-week adaptation period, mice were killed by cervical dislocation. Whole stomach and intestine of each mouse were quickly excised and placed in an ice-cold Hank's buffer saline solution. Circular muscle strips (10 mm in length and 0.2 mm in width) were cut from the distal antrum region and longitudinal strips from jejunum, ileum, and proximal colon. Muscle preparations were suspended vertically in an organ bath filled with Krebs solution (NaCl, 117 mM; KCl, 4.7 mM; MgCl_2_, 1.2 mM; NaH_2_PO_4_, 1.2 mM; NaHCO_3_, 25 mM; CaCl_2_, 2.5 mM; and glucose, 11 mM) warmed at 37°C and gassed with 95% O_2_+5% CO_2_. Muscle strips were stretched with a tension of 4–6 mN. Isometric contractions were continuously recorded by using isometric force transducers (No. TRI202PAD; Panlab, Cornellã, Spain) coupled to a computer equipped with the PowerLab 8/30 System and the Labchart data analysis software (AD Instruments, Spechbach, Germany).

Basal effects were assessed after a 30-min equilibration period. Muscle strips (*n* = 10 per condition) were treated with increasing concentrations of *M. officinalis* HLE (1, 5, 10, 25, and 50 mg/mL corresponding, respectively, to 0.077, 0.39, 0.77, 1.9, and 3.87 mg/mL of dry material and 0.004, 0.02, 0.04, 0.1, and 0.2 mg/mL of rosmarinic acid) or ethanol (at corresponding concentrations, *i.e*., 0.3, 1.5, 3, 7.5, and 15 mg/mL). Muscle strips were then washed in Krebs solution for 15 min and treated with nifedipine 10 *μ*M (myorelaxant positive control) after a 15-min equilibration period. The protocol for the assessment of spasmolytic effects was identical except steady-state muscle strips were precontracted with the muscarinergic agonist bethanechol (100 *μ*M) before treatment with *M. officinalis* HLE or ethanol then, after washing with Krebs solution, treated with bethanechol and nifedipine for control.

Frequency of spontaneous phasic contractions was evaluated for 2 min and contractile activity was assessed by measuring the area under the curve (AUC) for 2 min. These two parameters were assessed before and after addition of *M. officinalis* HLE or ethanol. Values are expressed as mean ± standard error of the mean. Graphs and statistical analysis were performed using GraphPad Prism 5.00 (GraphPad Software, Inc., La Jolla, CA, USA). Friedman's test followed by Dunn's test was used to analyze the dose–response effect of *M. officinalis* HLE or ethanol. Treatment effects were compared using two-way analysis of variance (ANOVA) for repeated measures followed by Bonferroni *post hoc* test. Nifedipine effects were analyzed with Wilcoxon test. Results were considered statistically significant when *P* < .05. All values were normalized to tissue weight and expressed in percentage compared with initial value before treatment with *M. officinalis* HLE or ethanol.

## Results

### Composition of *M. officinalis* HLE

**FIG. 1.**
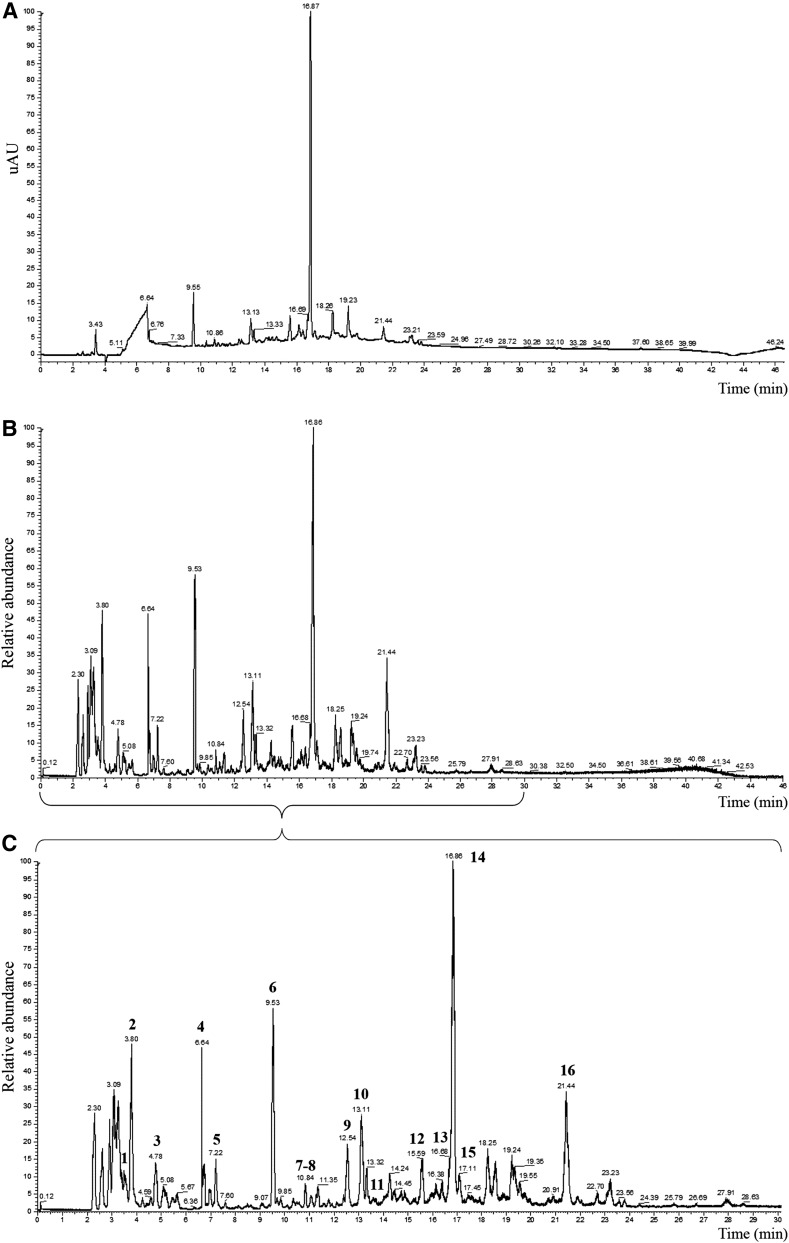
UHPLC chromatograms of *Melissa officinalis* HLE. **(A)** UV at 190–400 nm; **(B)** mass spectrum in the negative ionization mode; **(C)** enlargement showing the 16 peaks identified. HLE, hydroethanolic leaf extract; UHPLC, ultra-high performance liquid chromatography; UV, ultraviolet.

*M. officinalis* HLE phytochemical profile was determined by liquid chromatography/mass spectrometry (LC/MS) and LC/MS^2^ analyses in the negative ionization mode. Major compound was rosmarinic acid (Fig. 1; Table 1, signal 14, M-H^−^: 359.0767). Several caffeic and rosmarinic acid derivatives such as danshensu (Fig. 1; Table 1, signal 6), 3′-O-(8′′-Z-caffeoyl)-rosmarinic acid (signal 15), ethyl caffeate (signal 16), and flavonoids such as luteolin 3′-O-*β*-d-glucuronide (signal 13) were also identified.

**Table 1.** Compounds Identified in *Melissa officinalis* Hydroethanolic Leaf Extract in the Negative Ionization Mode with Ultra-High Performance Liquid Chromatography MS and MS^2^

*Peak*	*Retention time (min)*	*Molecular ion [M-H]^−^ (m/z)*	*Formula*	*MS^2^ (m/z)*	*Compounds*	*Reference*
1	3.59	149.0086	C_4_H_6_O_6_	(149)/87/72/59/103	Tartaric acid	Standard
2	3.80	191.0556	C_7_H_12_O_6_	(191)/85/127	Quinic acid	MassBank of North America Quinate^25^
3	4.78	133.0137	C_4_H_6_O_5_	115/71/(133)/89	Malic acid	^26^
4	6.64	191.0192	C_6_H_8_O_7_	111/87/85/(191)	Citric acid	MassBank of North America Citrate^27^
5	7.22	117.0188	C_4_H_6_O_4_	73/(117)/99	Succinic acid	MassBank of North America Succinate^28^
6	9.53	197.0450	C_9_H_10_O_5_	72/135/123/179	Danshensu	^29^
7	10.84	311.0403	C_13_H_12_O_9_	149/179/135/87	Caftaric acid	^30^
8	10.84	179.0345	C_9_H_8_O_4_	135	Caffeic acid (isomere)	Standard
9	12.54	137.0239	C_7_H_6_O_3_	(137)	Salicylic acid	^31^
10	13.11	179.0345	C_9_H_8_O_4_	135	Caffeic acid	Standard
11	13.71	537.1033	C_27_H_22_O_12_	295/179/135/121/(493)	Lithospermic acid A	^32^
12	15.59	473.0720	C_22_H_18_O_12_	149/179/135 (311/293)	Chicoric acid	^26^
13	16.68	461.0720	C_21_H_18_O_12_	285	Luteolin 3′-O-*β*-d-glucuronide	MassBank of North America Luteolin-7-O-glucoside^33^
14	16.86	359.0767	C_18_H_16_O_8_	161/197/179/135	Rosmarinic acid	Standard
15	17.11	537.1033	C_27_H_22_O_12_	161/135/359/179/197	3′-O-(8′′-Z-caffeoyl) rosmarinic acid	^34^
16	21.44	207.0663	C_11_H_12_O_4_	(207)/135/179/161/134/133	Ethyl caffeate	^35^

MS, mass spectrometry; MS^2^, tandem mass spectrometry.

### Effects on GI tract motility

#### Antrum

Both in basal and precontracted conditions, effects of *M. officinalis* HLE on antrum were not significantly different from those of ethanol (vehicle; Table 2).

**Table 2.** Summary of the Effects of *Melissa officinalis* Hydroethanolic Leaf Extract on the Different Parts of the Gastrointestinal Tract

*GI part*	*Treatment*	*Basal effects (basal conditions)*		*Spasmolytic effects (precontracted conditions)*	
		*Frequency of spontaneous phasic contractions*	*Contractile activity (AUC)*	*Frequency of spontaneous phasic contractions*	*Contractile activity (AUC)*
Antrum	*M. officinalis* HLE (mg/mL)	↓ at 25 and 50	NS	NS	NS
	Ethanol (equiv. mg/mL)	↓ at 25 and 50	↓ at 5 and 10	↓ at 50	↓ at 25 and 50
	Comparison *M. officinalis*/ethanol	NS	NS	NS	NS
Jejunum	*M. officinalis* HLE (mg/mL)	↓ at 25 and 50	↓ at 10, 25 and 50	↓ at 25 and 50	↓ at 10, 25 and 50
	Ethanol (equiv. mg/mL)	NS	NS	NS	↓ at 10, 25 and 50
	Comparison *M. officinalis*/ethanol	↓ at 25 and 50	↓ at 10, 25 and 50	↓ at 50	↓ at 10, 25 and 50
Ileum	*M. officinalis* HLE (mg/mL)	↓ at 50	NS	↓ at 50	↓ at 10, 25 and 50
	Ethanol (equiv. mg/mL)	NS	↓ at 50	↑ at 25	↓ at 10, 25 and 50
	Comparison *M. officinalis*/ethanol	↓ at 25 and 50	NS	↓ at 50	NS
Colon	*M. officinalis* HLE (mg/mL)	–	↑	–	NS
	Ethanol (equiv. mg/mL)	–	↓ at 25 and 50	–	↓ at 25 and 50
	Comparison *M. officinalis*/ethanol	–	NS	–	NS

–, not tested; AUC, area under the curve; GI, gastrointestinal; HLE, hydroethanolic leaf extract; NS, not significant.

In basal conditions, both *M. officinalis* HLE and ethanol induced a significant decrease in the frequency of antrum contractions at 25 and 50 mg/mL (for HLE, doses correspond, respectively, to 1.9 and 3.87 mg/mL of dry material; Friedman's test: *P* = .0003 [*n* = 9] for *M. officinalis* HLE, *P* < .0001 [*n* = 7] for ethanol; Dunn's test: −94.6% ± 5.4% and −100% ± 0.0%, respectively, *P* < .05 vs. control [*i.e*. previous treatment] for both *M. officinalis* HLE and ethanol; data not shown) without any significant difference between two treatments (two-way ANOVA, *P* = .95; *M. officinalis* HLE *n* = 9; ethanol *n* = 7). There was no significant effect of *M. officinalis* HLE on the contractile activity of antrum (AUC), whereas ethanol significantly decreased AUC at corresponding concentrations of 5 and 10 mg/mL (Friedman's test: *P* = .0005 [*n* = 7]; Dunn's test: *P* < .05 vs. control). However, the difference between the two treatments was not significant.

In the precontracted antrum, there was no significant change in frequency of phasic contractions with *M. officinalis* HLE whatever the concentration (Friedman's test: *P* = .12 [*n* = 10]). Although ethanol induced a significant decrease in that frequency at corresponding concentration of 50 mg/mL (Friedman's test: *P* = .0002 [*n* = 10]; Dunn's test: *P* < .05 vs. control [*n* = 10]), the difference between two treatments was not significant (two-way ANOVA, *P* = .83; *M. officinalis* HLE *n* = 10; ethanol *n* = 10). Similar observations were made for the contractile activity (AUC): no effect of *M. officinalis*, significant effect of ethanol at corresponding concentrations of 25 and 50 mg/mL, and no difference between the two treatments.

Nifedipine significantly inhibited phasic contractions and AUCs in all conditions (for antrum and all GI parts).

#### Jejunum

**FIG. 2.**
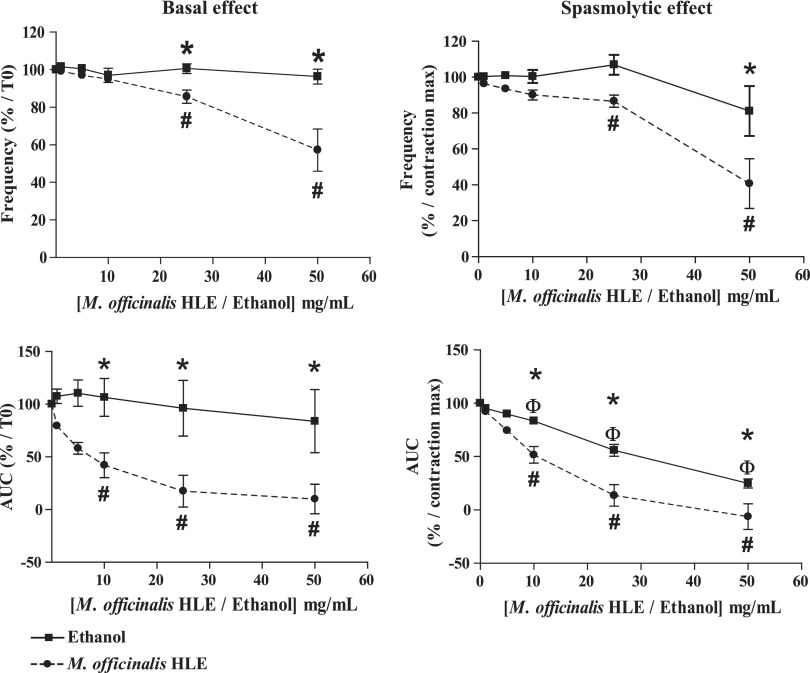
Effects of *M. officinalis* HLE on the jejunum. Friedman's test followed by Dunn's test were used to analyze the dose–response effect of *M. officinalis* HLE or ethanol and values that are significantly different from control (*i.e*., previous treatment) are indicated respectively by # and Φ. Treatment effects were compared using two-way ANOVA for repeated measures followed by Bonferroni *post hoc* test and values that are significantly different are indicated by *. ANOVA, analysis of variance; AUC, area under the curve.

**FIG. 3.**
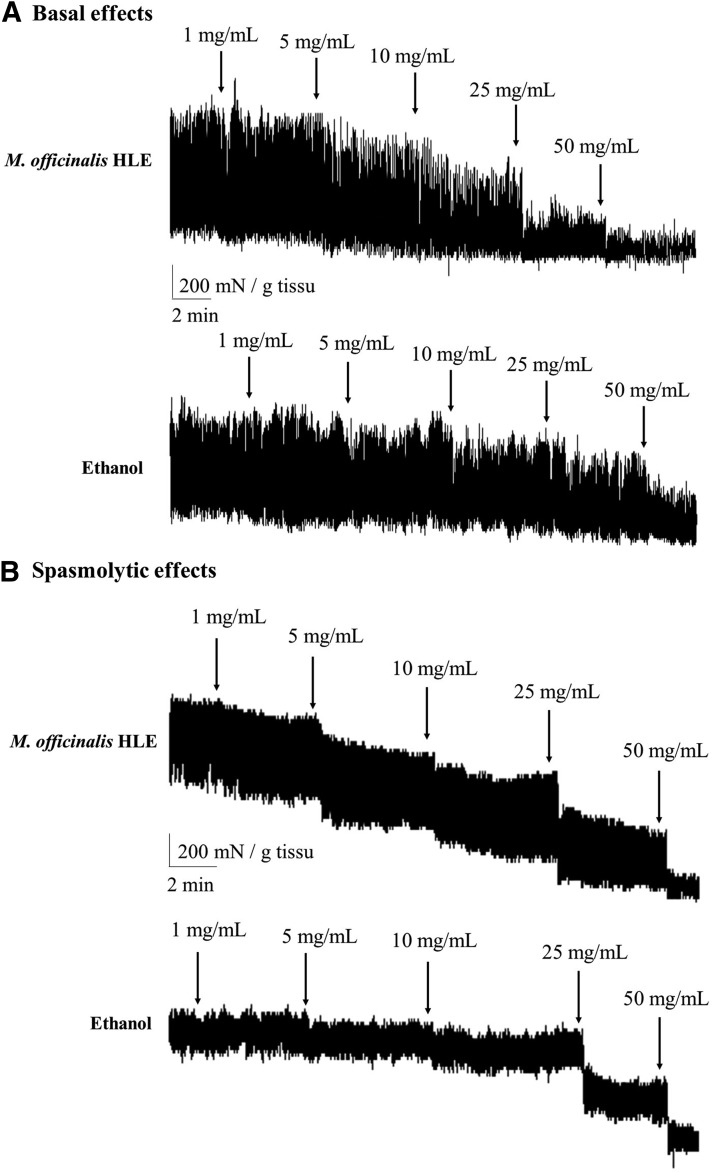
Typical recording of *M. officinalis* HLE and ethanol dose–response inhibition of contraction of isolated jejunum longitudinal muscle strip from mice under basal conditions **(A)** and precontracted conditions **(B)**.

In basal conditions (Table 2, Figs. 2 and 3A), *M. officinalis* HLE significantly decreased the frequency of spontaneous phasic contractions of jejunum at 25 and 50 mg/mL (corresponding, respectively, to 1.9 and 3.87 mg/mL of dry material; Friedman's test: *P* < .0001 [*n* = 9]; Dunn's test: −14.2% ± 3.1% and −48.5% ± 11.6%, respectively, *P* < .05 vs. control [*i.e*. previous treatment]), whereas no decrease was observed with ethanol (Friedman's test: *P* = .23 [*n* = 10]). The difference between *M. officinalis* and ethanol was statistically significant at 25 and 50 mg/mL (two-way ANOVA, *P* = .0015; Bonferroni test, *P* < .05; *M. officinalis* HLE *n* = 9; ethanol *n* = 10). With regard to the contractile activity, a significant AUC reduction was observed with *M. officinalis* HLE at 10, 25, and 50 mg/mL (corresponding, respectively, to 0.77, 1.9, and 3.87 mg/mL of dry material; Friedman's test: *P* < .0001 [*n* = 9]; Dunn's test: −58.0% ± 11.8%, −82.6% ± 15.1%, and −90.1% ± 14.0%, respectively, *P* < .05 vs. control). The difference between the two treatments was statistically significant at 10, 25, and 50 mg/mL (two-way ANOVA, *P* = .011; Bonferroni test, *P* < .05; *M. officinalis* HLE *n* = 9; ethanol *n* = 10).

Similarly, in precontracted conditions (Table 2, Figs. 2 and 3B), *M. officinalis* HLE significantly decreased frequency of spontaneous phasic contractions of jejunum at 25 and 50 mg/mL (corresponding, respectively, to 1.9 and 3.87 mg/mL of dry material; Friedman's test: *P* < .0001 [*n* = 10]; Dunn's test: −13.5% ± 3.4% and −59.3% ± 13.9%, respectively, *P* < .05 vs. control [*i.e*., previous treatment]), whereas no decrease was reported with ethanol (Friedman's test: *P* = .34 [*n* = 10]). The difference between two treatments was statistically significant at 50 mg/mL (two-way ANOVA, *P* = .011; Bonferroni test, *P* < .05; *M. officinalis* HLE *n* = 10; ethanol *n* = 10). As for the contractile activity, both *M. officinalis* HLE and ethanol induced a significant decrease in AUC at 10, 25, and 50 mg/mL (for HLE, doses correspond, respectively, to 0.77, 1.9, and 3.87 mg/mL of dry material; Friedman's test: *P* < .0001 [*n* = 10], Dunn's test: *P* < .05 for both *M. officinalis* HLE and ethanol; −48.4% ± 7.7%, −86.3% ± 10.1%, and −106.2% ± 11.9%, respectively, for *M. officinalis* HLE). AUC decrease observed with *M. officinalis* HLE was significantly greater than that with ethanol, at corresponding concentrations of 10, 25, and 50 mg/mL (two-way ANOVA, *P* = .0021; Bonferroni test, *P* < .05; *M. officinalis* HLE *n* = 10; ethanol *n* = 10).

#### Ileum

**FIG. 4.**
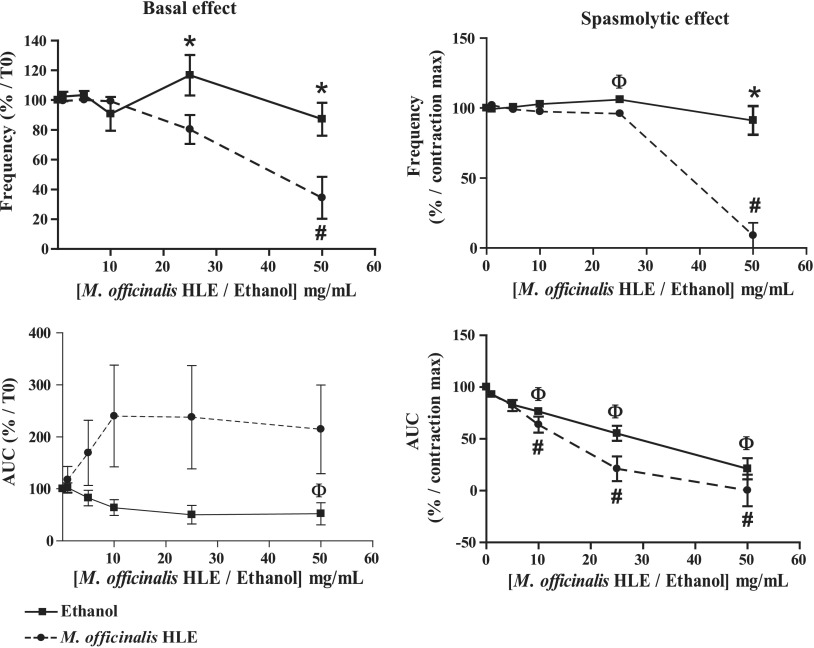
Effects of *M. officinalis* HLE on the ileum. Friedman's test followed by Dunn's test were used to analyze the dose–response effect of *M. officinalis* HLE or ethanol and values that are significantly different from control (*i.e*., previous treatment) are indicated respectively by # and Φ. Treatment effects were compared by using two-way ANOVA for repeated measures followed by Bonferroni *post hoc* test and values that are significantly different are indicated by *.

In basal conditions (Table 2 and Fig. 4), *M. officinalis* HLE significantly decreased the frequency of spontaneous phasic contractions of ileum at 50 mg/mL (corresponding to 3.87 mg/mL of dry material; Friedman's test: *P* < .0001 [*n* = 10]; Dunn's test: −65.6% ± 14.1% *P* < .05 vs. control [*i.e*., before treatment]). In contrast, there was no significant decrease with ethanol (Friedman's test: *P* = .057 [*n* = 9]). The difference between *M. officinalis* HLE and ethanol was statistically significant at 25 and 50 mg/mL (two-way ANOVA, *P* = .0009; Bonferroni test, *P* < .05; *M. officinalis* HLE *n* = 10; ethanol *n* = 9). *M. officinalis* HLE had no effect on contractile activity of ileum (Friedman's test: *P* = .81 [*n* = 10]), whereas ethanol decreased AUC at corresponding concentration of 50 mg/mL (Friedman's test: *P* = .0002 [*n* = 10]; Dunn's test: *P* < .05 vs. control). The difference between two treatments was not significant (two-way ANOVA, *P* = .092; *M. officinalis* HLE *n* = 10; ethanol *n* = 10).

In precontracted conditions (Table 2 and Fig. 4), frequency of spontaneous phasic contractions of ileum was significantly decreased with *M. officinalis* HLE at 50 mg/mL (corresponding to 3.87 mg/mL of dry material; Friedman's test: *P* < .0001 [*n* = 9]; Dunn's test: −91.9% ± 8.1%, *P* < .05 vs. control [*i.e*., before treatment]) and ethanol at corresponding concentration of 25 mg/mL (Friedman's test: *P* = .0008 [*n* = 10]; Dunn's test: *P* < .05). The difference between the two treatments was statistically significant at 50 mg/mL (two-way ANOVA, *P* < .0001; Bonferroni test, *P* < .05; *M. officinalis* HLE *n* = 9; ethanol *n* = 10). As for contractile activity, both *M. officinalis* HLE and ethanol induced a significant decrease in the AUC at 10, 25, and 50 mg/mL (for HLE, doses correspond, respectively, to 0.77, 1.9, and 3.87 mg/mL of dry material; Friedman's test: *P* < .0001, Dunn's test: *P* < .05 vs. control for both *M. officinalis* HLE [*n* = 9] and ethanol [*n* = 10]; −36.2% ± 7.8%, −78.8% ± 12.0%, and −99.7% ± 15.6%, respectively for *M. officinalis* HLE). The decrease in AUC observed with *M. officinalis* HLE was not significantly different from that with ethanol (two-way ANOVA, *P* = .11; *M. officinalis* HLE *n* = 9; ethanol *n* = 10).

#### Proximal colon

Although *M. officinalis* HLE significantly increased colon contractile activity in basal conditions (Table 2; Friedman's test: *P* < .045 vs. control [*i.e*., previous treatment] [*n* = 9]), ethanol significantly decreased the AUC at corresponding concentrations of 25 and 50 mg/mL (Friedman's test: *P* < .0001 [*n* = 10]; Dunn's test: *P* < .05), the difference between the two treatments was not significant (two-way ANOVA, *P* = .25; *M. officinalis* HLE *n* = 9; ethanol *n* = 10; data not shown).

In precontracted conditions, *M. officinalis* HLE had no effect on the contractile activity of proximal colon (Friedman's test: *P* = .25 [*n* = 9]), whereas ethanol significantly decreased AUC at 25 and 50 mg/mL (Friedman's test: *P* < .0001 [*n* = 10]; Dunn's test: *P* < .05). The difference between two treatments was not significant (two-way ANOVA, *P* < .77; *M. officinalis* HLE *n* = 9; ethanol *n* = 10).

## Discussion

Our results show that the HLE of *M. officinalis* tested has site- and dose-dependent effects on the contractile activity of the GI tract that do not depend on its vehicle (ethanol). In particular, *M. officinalis* HLE affected motility response in jejunum and ileum, whereas it had no effect on antrum and colon.

As for the small intestine, *M. officinalis* HLE inhibited AUC and contractile frequency at low concentrations for which ethanol had no effect. In addition, at higher concentration, the amplitude of effects was also significantly higher than that observed with ethanol (when it had an effect). Whether these changes observed *ex vivo* impact intestinal transit remains to be assessed *in vivo*. However, this decrease in contractile activity observed both under basal condition and after precontraction could lead to slower intestinal transit. Conversely, spasmolytic activity could contribute to reduce intestinal sensitivity. Consistent with our findings, inhibitory effects of *M. officinalis* on ileum have been reported in several studies.^6,7,11^ In brief, an ethanol extract of *M. officinalis* folium inhibited histamine-induced contractions of guinea pig ileum, whereas an aqueous extract was inactive.^11^ Interestingly, a hydroethanolic extract (30% ethanol dose unknown) also decreased histamine-induced contractions and amplitude of spontaneous contractions in the guinea pig ileum in comparison with solvent control.^7^ However, this extract had no effect on amplitude and frequency of slow waves in circular smooth muscle of mouse small intestine, suggesting other mechanisms of action underlying functional effects of the extract (such as altering intracellular calcium homeostasis).^10^ In addition, no antispasmodic activity was observed for a hydroethanolic extract (30% ethanol; 2.5 and 10 mL/L) in the guinea pig ileum.^6^ Finally, our study also extended spasmolytic activity of *M. officinalis* HLE to the jejunum that seems to be more sensitive to the extract both under basal and precontracted conditions.

Of interest, *M. officinalis* HLE had no effects on antrum and colon contractile activity but seemed to antagonize the effects of ethanol. As several studies showed that gastric emptying was slowed by ethanol (*e.g*., Jian *et al*. ^12^), our results suggest that *M. officinalis* could prevent ethanol-induced slowing of gastric emptying. In contrast to our findings, a *M. officinalis* extract used as an ethanol-free lyophilisate induced a small but significant contractile response in the antrum.^8^ In our study, *M. officinalis* HLE had no significant effects on proximal colon motility; these results are similar to those of Sibaev *et al.* who reported that an ethanolic extract of lemon balm had no effect on amplitude and frequency of slow waves in the colon.^9^ The underlying mechanisms of the organ-specific effects of *M. officinalis* reported in our study remains currently unknown. It could however reflect differences in properties of smooth muscle cells and/or interstitial cells of Cajal between different gut segments. These differences are probably not because of region-specific differences in the neurochemical phenotype of the enteric nervous system as previous studies have shown that SWT 5 (that contains among others *M. officinalis*) effects were not modified by synaptic transmission blockers.^13^

Main compounds found in *M. officinalis* HLE were rosmarinic acid, and other phenolic compounds, as previously reported in literature.^3,14,15^ Our phytochemical analysis also revealed hydroxyl cinnamic acids such as caffeic acid and derivatives, and of several compounds containing carboxylic acid function including quinic, citric, and salicylic acids. Luteolin 3′-O-*β*-d-glucuronide was also found; this substance was previously reported as the major flavone present in *M. officinalis.*^14,15^ Which individual component or group of components of *M. officinalis* influences GI activity and is responsible for which specific effect will have to be answered in future investigations. However, effects of *M. officinalis* are mainly attributed to phenolic compounds and its essential oil.^3^ Studies performed with the essential oil of *M. officinalis* showed a myorelaxant effect on different parts of the small intestine in various species.^16–19^ However, as shown in our phytochemical analysis our extract does not contain essential oil (no terpenes were detected). Therefore, effects observed cannot be attributed to essential oil, but are likely because of rosmarinic acid and/or other phenolic compounds. An aqueous extract of *M. officinalis* was reported to have a vasorelaxant effect on the rat isolated thoracic aorta.^20^ Rosmarinic acid was the most abundant compound in this extract, and the authors confirmed that the compound exerted a vasorelaxant effect. Rosmarinic acid was also found to have spasmolytic effects on rat uterus.^21^ However, rosmarinic acid (from thyme) had no antispasmodic activity in a preconstricted rat smooth muscle trachea model; the effect observed was attributed to luteolin.^22^ Therefore, luteolin 3′-O-*β*-d-glucuronide present in our extract could be, at least partly, at the origin of observed effects.

Overall, the effects of *M. officinalis* HLE tested on gut motility are site and dose dependent. Whether the effects observed *ex vivo* have an impact on intestinal transit will have to be assessed *in vivo*. These effects could be because of an action of phenolic compounds but further experiments will have to confirm this hypothesis. Benefits of *M. officinalis* could be related to site-dependent spasmolytic actions, besides its previously described anti-inflammatory, antinociceptive, and prosecretory action.^3,23,24^
